# Hydrogel Derived from Glucomannan-Chitosan to Improve the Survival of *Lactobacillus acidophilus* FNCC 0051 in Simulated Gastrointestinal Fluid

**DOI:** 10.1155/2022/7362077

**Published:** 2022-12-14

**Authors:** Veriani Aprilia, Agnes Murdiati, Pudji Hastuti, Eni Harmayani

**Affiliations:** ^1^Department of Nutrition Science, Faculty of Health Sciences, Universitas Alma Ata, Jl. Brawijaya 99, Tamantirto, Yogyakarta 55183, Indonesia; ^2^Department of Food and Agriculture Product Technology, Faculty of Agricultural Technology, Universitas Gadjah Mada, Jl. Flora, Bulaksumur, Yogyakarta 55281, Indonesia

## Abstract

The probiotic encapsulating hydrogel derived from porang (*Amorphophallus oncophyllus*) glucomannan and chitosan was investigated with regard to its encapsulation efficiency, physical properties, prebiotic activity, and survival under simulated gastrointestinal conditions. The hydrogel's encapsulation efficiency was improved by varying the number of the *Lactobacillus acidophilus* FNCC 0051, which also served to increase the diameter (2-3 mm), polydispersity index (1.23–1.65), positive zeta potential, whiteness, and brightness of the hydrogel. Moreover, the hydrogel's prebiotic activity score was higher than that of inulin after 24 h of incubation, reflecting its role as a cell encapsulant, particularly when it comes to maintaining cells during exposure to simulated gastrointestinal fluid. The cell viability increased from 86% to 100% when immersed in intestinal juice, which is comparable to the increase achieved using alginate and konjac glucomannan hydrogels. Future animal studies are required to determine the cell viability in actual gastrointestinal conditions and assess the health effects of the hydrogel.

## 1. Introduction

Glucomannan is a functional polysaccharide that can be extracted from *Amorphophallus* tubers. While the glucomannan obtained from *Amorphophallus konjac* has a number of popular and commercial uses, several research groups are currently investigating the potential of glucomannan derived from other sources. *Amorphophallus oncophyllus*, which is commonly known as porang, is a local glucomannan source in Indonesia [1, 2]. It has several characteristics that differ from those of konjac, including mannose/glucose molar ratio, degree of polymerization, and degree of acetylation, leading it to exhibit different solubility, viscosity, water-holding capacity, and gelation properties [1, 2]. Therefore, the applications of porang may also differ depending on the function.

A hydrogel is a kind of technological glucomannan product that leverages its gelation properties. Hydrogels are formed through interactions between glucomannan and other polymers that lead to the formation of a three-dimensional polymeric network [3]. This characteristic results in hydrogels exhibit potential as encapsulants. A previous study used a hydrogel created by crosslinking konjac, glucomannan, and chitosan, which was found to have many advantages, including natural formation without the need for a crosslinker, self-assembly, tolerance to different pH levels, and demonstrable ability to encapsulate drugs, proteins, and enzymes [4, 5]. A similar study involving hydrogels formed by means of the interaction between porang glucomannan and chitosan investigated the production of the primary carboxymethyl glucomannan material, the compatibility of the substitution degree of the carboxymethyl glucomannan involved in the hydrogel formation, the effect of the polymer concentration on the glucomannan properties, and the application in relation to probiotic encapsulation [6–8]. The key innovation of the study was the use of porang, which has characteristics that differ from those of other glucomannan sources, such as the solubility, viscosity, water-holding capacity, degree of polymerization, degree of acetylation, purity, and X-ray diffraction (XRD) pattern [1, 2]. The other differences include the type of modification used (carboxymethylation) and the use of the hydrogel as a probiotic encapsulant. By contrast, prior studies made use of the oxidation method [5] and encapsulated drugs, proteins, and enzymes [4, 5]. The use of carboxymethyl konjac glucomannan-chitosan as a probiotic encapsulant was recently studied, but it was combined with a calcium-alginate hydrogel bead system [9]. They were also found to be used as a secondary emulsion to carry curcumin [10].

However, given that living cells have different characteristics to inanimate compounds, the role of this new hydrogel in encapsulating probiotics needs to be further studied. Indeed, the new capsules should ensure the survival of the probiotics during food processing and storage, in addition to ensuring sufficient delivery when consumed (>10^6^-10^7^ colony-forming units [CFU]/mL). Furthermore, the capsules need to allow the probiotics to reach the lower gastrointestinal tract if they are to have a beneficial effect on humans. Thus, the survival of the capsules during gastrointestinal digestion and their ability to increase probiotic growth in the colon are important.

We previously studied the properties of the hydrogel produced in the different glucomannan concentration and evaluated its probiotic encapsulation efficiency, also its role in protecting cells during pasteurization and cold storage [8]. Encapsulation efficiency could not only be improved by varying the concentration of added polymer but also added core [11]. The impact of probiotic cells number as the core on the encapsulation efficiency and the properties of the hydrogel in this work remain unexplored. The present study sought to improve the probiotic encapsulation efficiency by varying the number of cells and to evaluate the hydrogel's physical properties. It also examined the ability of the hydrogel to maintain probiotics during simulated gastrointestinal exposure and its potency as a prebiotic.

## 2. Materials and Methods

### 2.1. Materials

The primary material used in this study was glucomannan derived from porang tubers (*A. oncophyllus*), which was obtained from the Faculty of Agricultural Technology, Universitas Gadjah Mada (Yogyakarta, Indonesia). The carboxymethylation of the glucomannan was performed using sodium chloroacetate, as previously described [7]. The utilized chitosan, which had a degree of deacetylation of 85%–89%, meaning that it met established food quality criteria, was obtained from PT Biotech Surindo (Cirebon, West Java, Indonesia).

### 2.2. Preparation of the *Lactobacillus acidophilus* FNCC 0051 Cells

The *L. acidophilus* FNCC 0051 cells used in this study were obtained from the Food and Nutrition Culture Collection (FNCC) of the Laboratory of Food Microbiology, Center for Food and Nutrition Studies, Universitas Gadjah Mada. The cells, which were stored in a skim milk-glycerol suspension, were rejuvenated in de Man, Rogosa, and Sharpe (MRS) broth at 37°C overnight and then grown twice. Subsequently, the cell biomass was harvested by means of centrifugation at 2400 g for 9 min at 4°C and then rinsed with saline solution.

### 2.3. Production of the Hydrogel and Determination of its Encapsulation Efficiency

The hydrogel was created by mixing porang glucomannan with chitosan using the complex coacervation method [8]. The encapsulation of the probiotics in the hydrogel was performed using three different cell numbers, namely 8 log CFU/mL, 9 log CFU/mL, and 10 log CFU/mL. The cells were mixed with glucomannan prior to the start of the coacervation process. The hydrogel's encapsulation efficiency was determined by releasing the cells trapped within it using a buffer solution at pH 8 and 37°C for 24 h [7]. The released cells were then grown in MRS agar to allow for the enumeration of the total viable cells. To calculate the encapsulation efficiency, the total viable cell number was divided by the number of initial cells added to the hydrogel mixture [12].

### 2.4. Determination of the Hydrogel's Properties

#### 2.4.1. Particle Size, Polydispersity Index, and Zeta Potential

The particle size was estimated based on the hydrogel's diameter and simultaneously measured on the basis of the polydispersity index using a particle size analyzer (SZ-100 series; Horiba, Kyoto, Japan). The hydrogel's zeta potential was measured using a Nano ZS Zetasizer (v.6.20; Malvern Instruments Ltd., Malvern, UK).

#### 2.4.2. Color

The hydrogel was freeze-dried and ground prior to the color measurement. The redness (*a*^*∗*^), yellowness (*b*^*∗*^), and lightness (*L*^*∗*^) values were determined using a CR200 chroma meter (Minolta, Osaka, Japan). The whiteness index was calculated as previously described [13].

#### 2.4.3. Crystallinity Percentage

The XRD of the hydrogel was determined using a LabX XRD-6000 diffractometer (Shimadzu, Kyoto, Japan) equipped with a Cu K*α* target at 40 kV and 30 mA, which had a scanning rate of 4°/min. The pattern was collected in the 2*θ* range between 3.02° and 90°. The crystallinity percentage (%) was calculated by dividing the area under the peaks by the total area under the curve [14].

### 2.5. Determination of the Prebiotic Activity Score

The prebiotic activity score was calculated by subtracting the ratio of probiotic cell growth with prebiotics and glucose from the ratio of enteric cell growth with prebiotics and glucose, as previously described [15]. The probiotic used was *L. acidophilus* FNCC 0051, whereas the enteric cells used were *Escherichia coli* FNCC 0091. The test was performed by adding 1% (volume/volume [v/v]) probiotic cells into MRS broth containing 2% (weight/volume [w/v]) glucose or prebiotic and adding 1% (v/v) enteric cells into M9 broth containing 2% (w/v) glucose or prebiotic. The cells were incubated at 37°C for 0 h, 24 h, and 48 h and then enumerated by means of the plate count method using MRS and nutrient agar. Each test was performed three times.

### 2.6. Determination of *L. acidophilus* FNCC 0051 Survival during Exposure to Simulated Gastrointestinal Conditions

The utilized simulated gastric and intestinal juices were prepared according to the method described by Xu et al. [16]. More specifically, the gastric juice was prepared by mixing 7 mL of pepsin in hydrochloric acid, 2 g of sodium chloride, and 1 M of sodium hydroxide. The intestinal juice was prepared by mixing 1% pancreatic powder, 6.8 g of potassium dihydrogen phosphate, and 77 mL of 0.2 N sodium hydroxide. Next, 1 g of either free or encapsulated cells (in hydrogel derived from porang glucomannan-chitosan, konjac glucomannan-chitosan, and calcium alginate) was mixed with 9 mL of simulated gastrointestinal juices and incubated at 37°C for 120 min. The samples were withdrawn at intervals of 0 min, 30 min, 60 min, and 120 min to reflect gastric juice digestion and 0 min, 60 min, 90 min, and 120 min to reflect intestinal juice digestion [17]. The hydrogel was then rinsed twice with acetate buffer. The cells were enumerated using the pour plate technique on MRS agar after 48 h of incubation. The number of viable cells following exposure was divided by the initial number of cells in order to determine the cell survival rate during exposure to simulated gastrointestinal conditions [12]. The hydrogel's appearance during exposure to simulated gastrointestinal conditions was observed using an optical BX51 microscope (Olympus Corp., Tokyo, Japan) and an OptiLab prodigital camera (PT Miconos, Indonesia).

## 3. Results and Discussion

### 3.1. Encapsulation Efficiencies of Hydrogels with Different Numbers of Cells

The encapsulation efficiencies of hydrogels with different numbers of initial cells are shown in Table 1. The data revealed that the encapsulation efficiencies of the hydrogels ranged between 44.37% and 85.03%. The highest encapsulation efficiency was achieved when 10 log CFU/mL of cells was added to the mixture, which exceeded the Food and Agricultural Organization of the United Nations (FAO) criteria for probiotic products (>6-7 log CFU/mL; [18]). Previous studies using different encapsulants obtained different encapsulation efficiencies. For instance, the encapsulation of *L. acidophilus* in hydrogel formed from sodium alginate and soy protein isolates achieved an encapsulation efficiency of 95%–98%, whereas the encapsulation of *Lactobacillus rhamnosus* and *Lactobacillus plantarum* in an emulsion achieved an encapsulation efficiency of 97%–99% [12, 19]. The differences in the achieved encapsulation efficiencies might reflect the different encapsulant types and encapsulation methods used [12]. We previously showed that the same ratio of glucomannan and chitosan affected the encapsulation efficiency due to the chemical bonding of both polymers as well as due to the difference in electrostatic values between the core and the polymer influencing the degree of cell entrapment [8].

### 3.2. Properties of the Hydrogels with Different Cell Numbers

**Figure 1:**
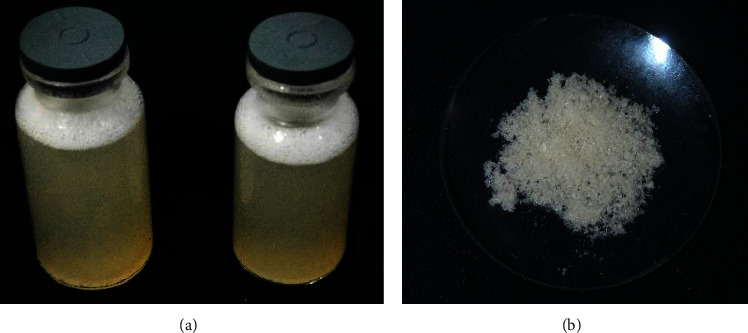
The appearance of hydrogels (a) before drying and (b) after the drying process.

The appearance of the hydrogels generated from glucomannan and chitosan containing *L. acidophilus* is shown in Figure 1. The polymer solution was clear before the encapsulation process, although it became turbid after the encapsulation process. This provided evidence of the formation of particles that influenced the turbidity of the solution. After the drying process, the hydrogels exhibited a shape similar to that of white cotton. The particle sizes and color values of the hydrogels will be explained.

The sizes of the hydrogels encapsulating *L. acidophilus* were found to be in the range of 0.7 *μ*m to 9 *μ*m, with most having a diameter of 2 *μ*m to 3 *μ*m (Table 2). Those hydrogels determined to be <100 *μ*m in diameter were classified as microgels. The cell concentration significantly influenced the hydrogels' particle size (*p*  <  0.05). In fact, the more cells encapsulated within a given hydrogel, the greater its diameter. The particle size was also correlated with the encapsulation efficiency (Table 1), as more cores could be trapped within larger hydrogel particles. The other factors found to influence the particle sizes were the concentration and viscosity of the solution [8, 12].

The polydispersity indexes of the hydrogel-encapsulated cells were all >1 (Table 2), indicating the broad distribution of particles of various sizes. Overall, the index began to change when the initial cell number was 10 log CFU/mL. Moreover, the greater the initial cell number, the higher the polydispersity index. This result contrasts with the result of a previous study that found the glucomannan concentration to not influence the polydispersity index [8].

The hydrogels' zeta potentials became more electropositive as the cell number increased from 8 to 9 log CFU/mL but then decreased as the cell number reached 10 log CFU/mL (Table 2). An increase in the number of cells should result in a reduction in the hydrogel's charge due to the positive charge of empty hydrogels and the negative charge of cells [8], including *L. acidophilus* [20]. The observed pattern might stem from the zeta potential being measured on the hydrogel's surface, meaning that it could have been affected by the pH of the surrounding environment [21].

The *L*^*∗*^, *b*^*∗*^, and whiteness values of the hydrogels increased after the addition of cells, whereas the *a*^*∗*^ value decreased (Table 3). The utilized instrument determined these values based on the reflection by the cells of a direct light beam from a chroma meter. Therefore, the more cells encapsulated within the hydrogel, the greater the reflection. Bacteria may also generate distinct shades of colors such as red. Based on the findings of a prior study, *Lactobacillus pluvialis* could reflect an orange color from the pigment of canthaxanthin [22]. This finding is in agreement with the present result, especially in terms of the increase in the *b*^*∗*^ value following the addition of *L. acidophilus.*

**Figure 2:**
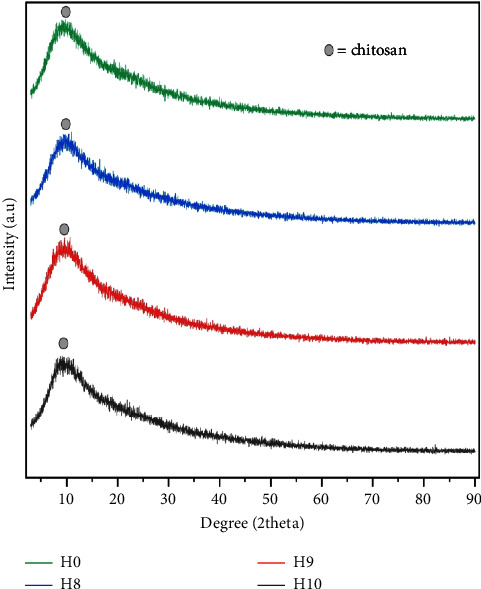
X-ray diffractogram for H0 (hydrogel without *L. acidophilus*), H8, H9, and H10 (hydrogels with *L. acidophilus* at numbers of 8 log CFU/mL, 9 log CFU/mL, and 10 log CFU/mL, respectively).

The XRD spectra represent the interaction between the diffraction intensity and the angle (Figure 2). Moreover, a crystalline state is indicated by the sharp diffraction peak, whereas an amorphous and solid state is indicated by the declivous peak [2]. The X-ray diffractogram patterns of all the hydrogels showed a very broad band at 2*θ* between 5° and 90°. In addition, all the hydrogels exhibited nearly identical highest peaks at around 2*θ* 7.06°–10.46°, 7.62°–11.00°, 7.48°–10.94°, and 7.16°–11.20° for those hydrogels without cells and with cells at numbers of 8 log CFU/mL, 9 log CFU/mL, and 10 log CFU/mL, respectively. These results differ from those concerning porang glucomannan, which exhibited its highest peaks at around 19°-20° and 35° [2]. However, there was a small peak in all the samples at around 2*θ* 10.5°, indicating the presence of chitosan [23]. This observation suggests that the mixture of glucomannan hydrogel and cells strengthened the associated chemical interaction, which is consistent with previous Fourier-transform infrared spectroscopy (FTIR) findings [8]. It also suggests that some chitosan did not interact with glucomannan. A prior study reported that Schiff's crosslinking between glucomannan aldehyde groups and chitosan amino groups could suppress the chitosan's crystalline state, which is usually strengthened by the hydrogen bond between the amino and hydroxyl groups [23]. We also found evidence of low crystallinity, with values of 26%, 25%, 17%, and 21% being determined for the hydrogels without cells and with cells at numbers of 8 log CFU/mL, 9 log CFU/mL, and 10 log CFU/mL, respectively. The addition of *L. acidophilus* appeared to have no effect on the diffraction peak, indicating that the entrapment of microbes within the hydrogel did not affect the interaction between glucomannan and chitosan.

### 3.3. Prebiotic Activity of the Hydrogels

The *L. acidophilus* and *E. coli* cell density increased during 0 h, 24 h, and 48 h of incubation in the presence of carbohydrates, glucose, inulin, and hydrogel (Table 4). Both bacteria showed no significant increase in almost all the carbohydrates, except for *L. acidophilus* with inulin and *E. coli* with glucose. These data suggest that only inulin is able to specifically stimulate the growth of good bacteria and suppress the growth of enteric bacteria, which is consistent with its widespread use as a commercial prebiotic.

**Figure 3:**
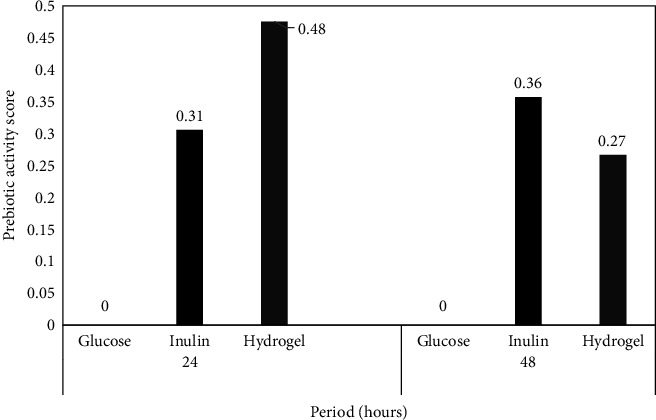
Prebiotic activity score of *L. acidophilus* FNCC 0051 on glucose, inulin, and hydrogel.

The prebiotic potential of the hydrogel was compared with that of inulin on the basis of the prebiotic activity scores (Figure 3). The prebiotic activity score of the hydrogel was higher than that of inulin after 24 h of incubation, although it was reduced after 48 h, suggesting that the hydrogel was the preferred energy source for the cells. This result is consistent with the XRD findings, which confirmed the hydrogel to have an amorphous state and no long-range order, making it easier to digest. Moreover, the amount of carbohydrates will decrease with time. By contrast, the known prebiotic inulin [24] required a longer time to be available for the bacteria due to its long polymeric carbon chains-that is, chains of around 2–60 molecules [25].

### 3.4. Cell Survival during Exposure to Simulated Gastrointestinal Conditions

#### 3.4.1. Cell Survival during Exposure to Gastric Juice

**Figure 4:**
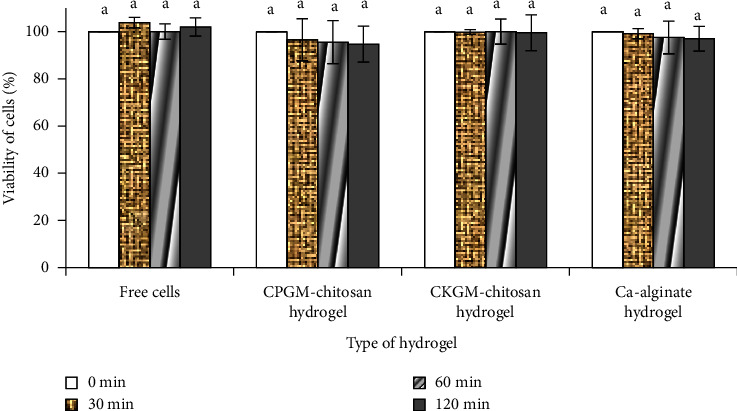
*L. acidophilus* FNCC 0051 viability during exposure to gastric juice for 120 min. Key: a, *p*  <  0.05, CPGM: carboxymethyl porang glucomannan, and CKGM: carboxymethyl konjac glucomannan.

The *L. acidophilus* showed good viability during exposure to gastric juice at pH 2, whether in its free form or when encapsulated in hydrogel (Figure 4). Generally, the growth of lactic acid bacteria is optimum at pH 6-7 (close to neutral pH). Some metabolic reactions change when the pH is <5 or <4.4. Indeed, some minerals will be lost at pH ≤2, while prolonged storage at a low pH will increase the risk of cell death [26]. Our results in this regard are consistent with those of previous studies [3, 12]. Furthermore, studies are required to determine the effect of solid or solid-enriched macronutrient foods with a longer transit time [27]. In addition, a shorter exposure time within the stomach enables cells to maintain homeostasis between the internal and external pH, which potentially influenced the good viability found in this study.

The present study also found that porang glucomannan-chitosan hydrogel might exhibit a similar ability to protect cells from the gastric environment as both konjac glucomannan-chitosan hydrogel and calcium-alginate hydrogel (*p*  >  0.05). This finding accords with the ability of alginate to protect *L. plantarum* [17] and *Lactobacillus rhamnosus* from this harsh environment over the course of 3 h of exposure [28].

**Figure 5:**
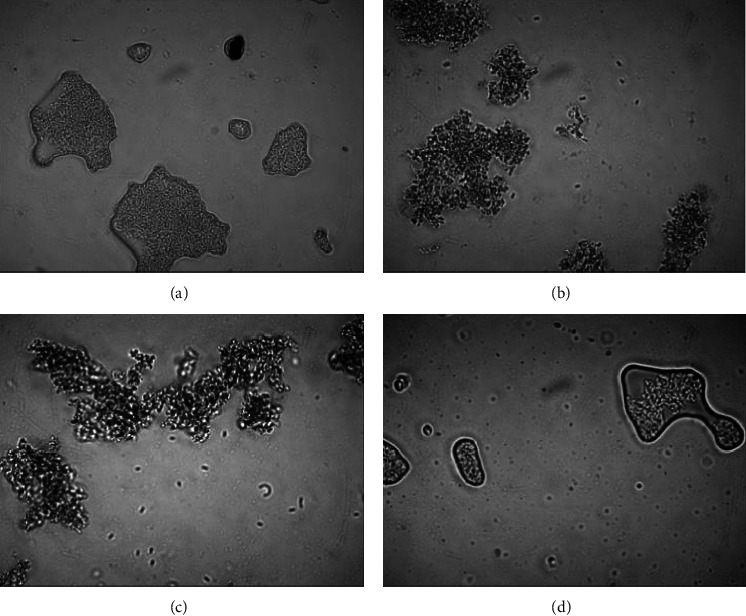
Microscopic appearance of hydrogel containing *L. acidophilus* FNCC 0051 (1300x magnification) during exposure to gastric juice for (a) 0 min, (b) 30 min, (c) 60 min, and (d) 120 min.

The hydrogel was stable in the simulated gastric juice throughout 120 min of exposure (Figure 5), which is consistent with the result of a previous swelling ratio study [8] that determined the hydrogel to not deswell at a pH <5. Deswelling causes the hydrogel to become smaller, which was previously thought to result in the release of cells from the hydrogel. However, the cells are still trapped in the hydrogel (Figure 5), which perhaps reflects the stronger electrostatic interaction between the glucomannan carbonyl group and chitosan amine group in an acid environment [8]. The cells remain in the hydrogel because this interaction maintains the core. Thus, deswelling could not be maximized, leading to only a small number of cells being released from the hydrogel. It is possible that some empty hydrogels will shrink to the extent that they are no longer visible after 60 min of exposure. These results are consistent with those of other studies using hydrogels made from oxidized glucomannan and chitosan to trap diclofenac drugs, which found <1% of cells to be released during exposure to simulated gastric fluid at pH 1.2 [5]. This shows that the hydrogel cores were not released when the hydrogel was exposed to low pH conditions.

#### 3.4.2. Cell Survival during Exposure to Intestinal Juice

**Figure 6:**
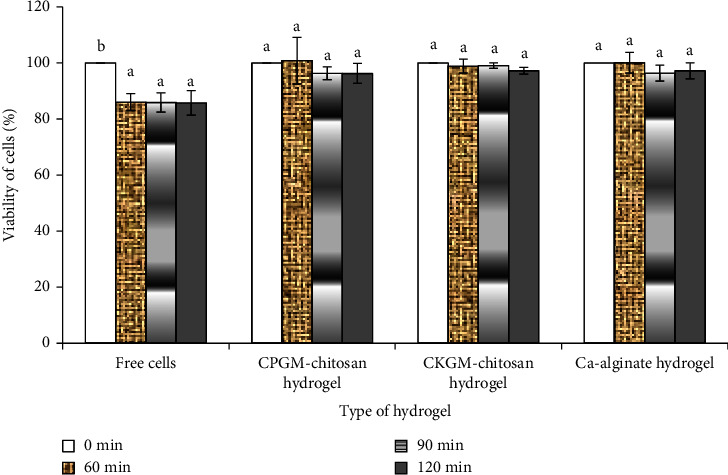
*L. acidophilus* FNCC 0051 cell viability during exposure to intestinal juice for 120 min. Key: a or b, *p*  <  0.05, CPGM: carboxymethyl porang glucomannan, and CKGM: carboxymethyl konjac glucomannan.

The viability of the free cells decreased significantly during exposure to intestinal juice for 60 min (Figure 6; *p*  <  0.05). Yet, the viability of the cells encapsulated in the hydrogel was maintained over 120 min of exposure, indicating that the encapsulation increased the viability of the *L. acidophilus*. A decrease in the number of free cells may reflect cell death, which can be caused by factors other than the pH of the medium. Priya et al. [20] reported that while bacteria showed good growth at pH 6.8, the presence of pancreatin (comprising amylase, trypsin, lipase, ribonuclease, and protease) damaged the encapsulation wall, thereby resulting in cell death.

Figure 6 indicates that the porang glucomannan hydrogel exhibited the same level of good protective effect as the konjac-chitosan glucomannan and calcium-alginate hydrogels. In this study, the alginate-based hydrogel was used for the purpose of comparison because it is widely used as an encapsulant due to its low price, good biocompatibility, and nontoxicity. A prior study found that the probiotic encapsulation of alginate increased the viability of the trapped cells when compared with the free cells during exposure to a simulated gastrointestinal condition [3]. Therefore, the porang-chitosan glucomannan hydrogel shows potential as a bacterial encapsulant.

**Figure 7:**
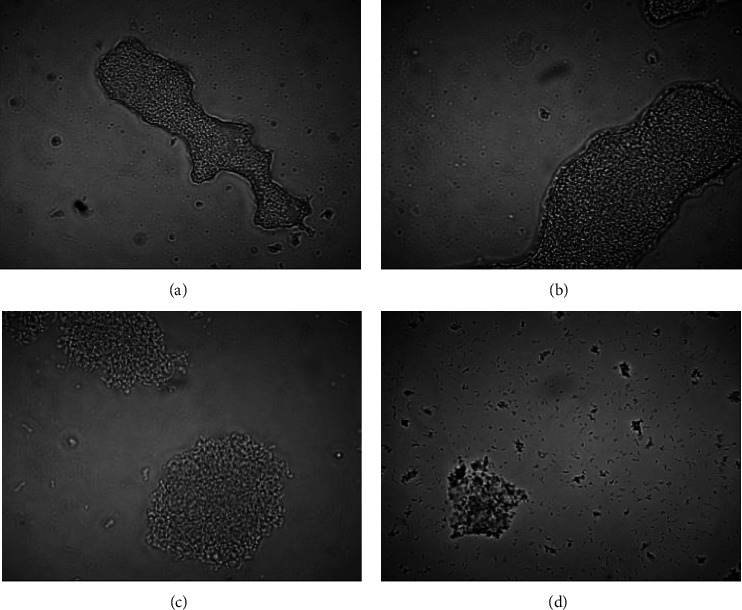
Microscopic appearance of hydrogel containing *L. acidophilus* FNCC 0051 (1300x magnification) during exposure to intestinal juice for (a) 0 min, (b) 60 min, (c) 90 min, and (d) 120 min.

The hydrogel's microscopic appearance was used to confirm the cell viability data. Here, the porang glucomannan-chitosan hydrogel remained stable for up to 2 h in the intestinal fluid. However, it was found to be larger after 60 min of exposure than after 0 min (Figure 7), potentially reflecting its swelling behavior at pH 6.8. We have previously shown that porang glucomannan-chitosan hydrogel begins to swell at pH >5 [8]. The swelling of the hydrogel was evident until it reached 90 min of exposure. Moreover, many small hydrogels and cells were visible in the solution after 120 min of exposure. The swelling weakened the interaction of the hydrogels, leading to some parts being dissolved, which resulted in both smaller hydrogels and the release of cells from the hydrogels. This result is consistent with that of another study that found konjac glucomannan-carboxymethyl chitosan hydrogel with a bovine serum albumin core to show greater core release at pH 7.4 than at pH 5 due to the swelling enlarging its pores [4]. This core release also occurred when a chitosan-oxidized glucomannan hydrogel was exposed to simulated intestinal fluid for 2–8 h [5].

## 4. Conclusions

The encapsulation of *L. acidophilus* in hydrogel made from glucomannan and chitosan was improved by varying the number of cells added. In fact, higher numbers were found to be associated with greater encapsulation efficiency, diameter (2-3 mm), polydispersity index (1.23–1.65), positive zeta potential, whiteness, and brightness. In addition, the hydrogel exhibited potential as a prebiotic, particularly after 24 h of incubation. Moreover, the hydrogel protected the encapsulated cells, maintaining them during exposure to simulated gastrointestinal fluid. Furthermore, the cell viability increased from 86% to 100% when the hydrogel was exposed to intestinal juice, which was comparable to the performance of the alginate and konjac glucomannan hydrogels. Furthermore, animal studies are required to determine the cell viability in actual gastrointestinal conditions and assess the health effects of the hydrogel.

## Figures and Tables

**Table 1:** Encapsulated cell numbers and hydrogel encapsulation efficiencies with different initial cell numbers.

Hydrogels with different cell numbers (log CFU/mL)	Cell number before encapsulation (log CFU/mL)	Cell number after encapsulation (log CFU/g)	Encapsulation efficiency (%)
8	9.39 ± 0.00	4.47 ± 0.18	44.37 ± 1.91^a^
9	9.56 ± 0.00	6.60 ± 0.13	65.83 ± 1.37^b^
10	10.10 ± 0.00	7.94 ± 0.21	85.03 ± 0.63^c^

Values represent the mean ± standard deviation (SD). Different superscript letters in the same column indicate significantly different results at the level of *p*  <  0.05.

**Table 2:** Particle sizes, polydispersity indexes, and zeta potentials of hydrogels with different initial cell concentrations.

Initial cell number (log CFU/mL)	Particle size (*μ*m)	Polydispersity index	Zeta potential (mV)
8	2.23 ± 0.11^a^	1.23 ± 0.17^a^	24.40 ± 0.75^b^
9	2.79 ± 0.19^b^	1.39 ± 0.04^ab^	32.28 ± 0.80^c^
10	3.41 ± 0.14^c^	1.65 ± 0.27^b^	14.58 ± 0.97^a^

Values represent the mean ± SD. Different superscript letters in the same column indicate significantly different results at the level of *p*  <  0.05.

**Table 3:** Color values of hydrogels with different initial cell numbers.

Initial cell number (log CFU/mL)	*L* ^*∗*^	*a* ^*∗*^	*b* ^*∗*^	Whiteness
Control	65.06 ± 0.12^a^	7.02 ± 0.09^c^	12.50 ± 0.08^a^	62.24 ± 0.15^a^
8	76.97 ± 0.32^b^	5.42 ± 0.01^b^	14.24 ± 0.11^c^	72.38 ± 0.21^b^
9	79.48 ± 0.33^c^	5.61 ± 0.07^b^	15.14 ± 0.01^d^	73.89 ± 0.25^c^
10	77.39 ± 0.23^b^	4.22 ± 0.23^a^	13.24 ± 0.13^b^	73.46 ± 0.30^c^

Values represent the mean ± SD. Different superscript letters in the same column indicate significantly different results at the level of *p*  <  0.05

**Table 4:** Density of *Lactobacillus acidophilus* FNCC 0051 and *Escherichia coli* cells in 10 log CFU/mL after 0 h, 24 h, and 48 h of incubation with prebiotics, inulin, hydrogel, and glucose.

Prebiotic	*Lactobacillus acidophilus*			*Escherichia coli*		
	0 h	24 h	48 h	0 h	24 h	48 h
Glucose	6.94 ± 1.32^a^	8.35 ± 0.81^a^	9.17 ± 0.01^b^	6.65 ± 0.92^a^	8.54 ± 0.09^ab^	9.29 ± 0.49^b^
Inulin	6.59 ± 0.19^a^	7.33 ± 0.49^ab^	8.48 ± 0.88^a^	9.53 ± 0.09^a^	7.59 ± 0.32^a^	8.47 ± 0.75^a^
Hydrogel	9.37 ± 0.10^a^	9.58 ± 0.46^a^	10.15 ± 0.21^b^	8.80 ± 1.13^a^	8.17 ± 0.86^a^	9.02 ± 2.18^a^

Values represent the mean ± SD. Different superscript letters in the same row indicate significantly different results at the level of *p*  <  0.05.

## Data Availability

The data used to support the findings of this study are included within the article.

## References

[1] Harmayani E., Aprilia V., Marsono Y. (2014). Characterization of glucomannan from Amorphophallus oncophyllus and its prebiotic activity in vivo. *Carbohydrate Polymers*.

[2] Yanuriati A., Marseno D. W., Rochmadi, Harmayani E. (2017). Characteristics of glucomannan isolated from fresh tuber of Porang (Amorphophallus muelleri Blume). *Carbohydrate Polymers*.

[3] Stasiak-Różańska L., Berthold-Pluta A., Pluta A. S., Dasiewicz K., Garbowska M. (2021). Effect of simulated gastrointestinal tract conditions on survivability of probiotic bacteria present in commercial preparations. *International Journal of Environmental Research and Public Health*.

[4] Du J., Dai J., Liu J. L., Dankovich T. (2006). Novel pH-sensitive polyelectrolyte carboxymethyl Konjac glucomannan-chitosan beads as drug carriers. *Reactive and Functional Polymers*.

[5] Korkiatithaweechai S., Umsarika P., Praphairaksit N., Muangsin N. (2011). Controlled release of diclofenac from matrix polymer of chitosan and oxidized konjac glucomannan. *Marine Drugs*.

[6] Aprilia V., Murdiati A., Hastuti P., Harmayani E. (2017a). Carboxymethylation of glucomannan from porang tuber (Amorphophallus oncophyllus) and the physicochemical properties of the product. *Pakistan Journal of Nutrition*.

[7] Aprilia V., Murdiati A., Hastuti P., Harmayani E. (2017b). Encapsulation of lactobacillus acidophilus FNCC 0051 in hydrogel using a complex coacervation of glucomannan and chitosan. *Research Journal of Microbiology*.

[8] Aprilia V., Murdiati A., Hastuti P., Harmayani E. (2021). The effect of carboxymethyl glucomannan concentration on the properties of glucomannan-chitosan hydrogel for lactobacillus acidophilus FNCC 0051 encapsulation. *Walailak Journal of Science and Technology*.

[9] Dinga X., Xu Y., Wang Y. (2022). Carboxymethyl konjac glucomannan-chitosan complex nanogels stabilized double emulsions incorporated into alginate hydrogel beads for the encapsulation, protection and delivery of probiotics. *Carbohydrate Polymers*.

[10] Wang L., Zhang X., Xiao J., Shi J. (2023). Effect of carboxymethyl konjac glucomannan coating on curcumin-loaded multilayered emulsion: stability evaluation. *Food Science and Human Wellness*.

[11] Li J., Hou X., Jiang L. (2022). Optimization and characterization of Sichuan pepper (Zanthoxylum bungeanum Maxim) resin microcapsule encapsulated with *β*-cyclodextrin. *Lebensmittel-Wissenschaft & Technologie*.

[12] Zeashan M., Afzaal M., Saeed F. (2020). Survival and behavior of free and encapsulated probiotic bacteria under simulated human gastrointestinal and technological conditions. *Food Sciences and Nutrition*.

[13] Akgün D., Ova Özcan D., Övez B. (2021). Optimization and characterization of cellulose nanocrystal production from aseptic tetra pak food packaging waste. *Journal of the Turkish Chemical Society, Section A: Chemistry*.

[14] Yazdani A., Hohne G. W. H., Misture S. T., Graeve O. A. (2020). A method to quantify crystallinity in amorphous metal alloys: a differential scanning calorimetry study. *PLoS One*.

[15] Zakaria Z., Amalinafitri Anis A., Napisah H., Shazila S. (2018). Prebiotic Activity Score of Breadfruit Resistant Starch (Artocarpus altilis), breadfruit flour, and inulin during in-vitro fermentation by pure cultures (Lactobacillus plantarum, and Bifidobacterium bifidum). *J. Agrobiotech*.

[16] Xu M., Gagne-Bourque F., Dumont M. J., Jabaji S. (2016). Encapsulation of Lactobacillus casei ATCC 393 cells and evaluation of their survival after freeze-drying, storage and under gastrointestinal conditions. *Journal of Food Engineering*.

[17] Rather S. A., Akhter R., Masoodi F., Gani A., Wani S. (2017). Effect of double alginate microencapsulation on in vitro digestibility and thermal tolerance of Lactobacillus plantarum NCDC201 and L. casei NCDC297. *LWT - Food Science and Technology*.

[18] Isa J. K., Razavi S. H. (2021). The behavior of lactobacillus casei as a potential probiotic in food carrier and simulated gastric juice. *Annals of R.S.C.B.*.

[19] Mahmoodi Pour H., Marhamatizadeh M. H., Fattahi H. (2022). Encapsulation of different types of probiotic bacteria within conventional/multilayer emulsion and its effect on the properties of probiotic yogurt. *Journal of Food Quality*.

[20] Priya A. J., Vijayalakshmi S. P., Raichur A. M. (2011). Enhanced survival of probiotic Lactobacillus acidophilus by encapsulation with nanostructured polyelectrolyte layers through layer-by-layer approach. *Journal of Agricultural and Food Chemistry*.

[21] Barbosa J. A. C., Abdelsadig M. S., Conway B. R., Merchant H. A. (2019). Using zeta potential to study the ionisation behaviour of polymers employed in modified-release dosage forms and estimating their pKa. *International Journal of Pharmaceutics X*.

[22] Venil C. K., Dufossé L., Renuka Devi P. (2020). Bacterial pigments: sustainable compounds with market potential for pharma and food industry. *Frontiers in Sustainable Food Systems*.

[23] Yu H., Lu J., Xiao C. (2007). Preparation and properties of novel hydrogels from oxidized konjac glucomannan cross-linked chitosan for in vitro drug delivery. *Macromolecular Bioscience*.

[24] Kamel D. G., Hammam A. R., Alsaleem K. A., Osman D. M. (2021). Addition of inulin to probiotic yogurt: viability of probiotic bacteria (Bifidobacterium bifidum) and sensory characteristics. *Food Sciences and Nutrition*.

[25] Samolińska W., Grela E. R. (2017). Comparative effects of inulin with different polymerization degrees on growth performance, blood trace minerals, and erythrocyte indices in growing-finishing pigs. *Biological Trace Element Research*.

[26] Hayek S. A., Ibrahim S. A. (2013). Current limitations and challenges with lactic acid bacteria: a review. *Food and Nutrition Sciences*.

[27] Müller M., Canfora E. E., Blaak E. E. (2018). Gastrointestinal transit time, glucose homeostasis and metabolic health: modulation by dietary fibers. *Nutrients*.

[28] Oberoi K., Tolun A., Altintas Z., Sharma S. (2021). Effect of alginate-microencapsulated hydrogels on the survival of lactobacillus rhamnosus under simulated gastrointestinal conditions. *Foods*.

[29] Collnot E. M., Ali H., Lehr C. M. (2012). Nano- and microparticulate drug carriers for targeting of the in fl amed intestinal mucosa. *Journal of Controlled Release*.

